# Novel Semi-Replicative Retroviral Vector Mediated Double Suicide Gene Transfer Enhances Antitumor Effects in Patient-Derived Glioblastoma Models

**DOI:** 10.3390/cancers11081090

**Published:** 2019-07-31

**Authors:** Mijeong Lee, Yeon-Soo Kim, Kyoungmin Lee, Moonkyung Kang, Hyemi Shin, Jeong-Woo Oh, Harim Koo, Donggeon Kim, Yejin Kim, Doo-Sik Kong, Do-Hyun Nam, Hye Won Lee

**Affiliations:** 1 Department of Health Science & Technology, Samsung Advanced Institute for Health Sciences & Technology (SAIHST), Sungkyunkwan University, Seoul 06351, Korea; 2 Institute for Refractory Cancer Research, Samsung Medical Center, Seoul 06351, Korea; 3 Graduate School of New Drug Discovery and Development, Chungnam National University, Daejeon 34134, Korea; 4 Department of Neurosurgery, Samsung Medical Center, Sungkyunkwan University School of Medicine, 81 Irwon-ro, Gangnam-gu, Seoul 06531, Korea; 5 Department of Anatomy and Cell Biology, Sungkyunkwan University School of Medicine, 2066 Seobu-ro, Suwon 16149, Korea; 6 Single Cell Network Research Center, Sungkyunkwan University, Suwon 16149, Korea

**Keywords:** glioblastoma, patient-derived glioblastoma stem-like cells, semi- and pseudotyped-retroviral replicating vector, dual suicide gene therapy, bystander effect

## Abstract

As glioblastomas are mostly localized infiltrative lesions, gene therapy based on the retroviral replicating vector (RRV) system is considered an attractive strategy. Combinations of multiple suicide genes can circumvent the limitations associated with each gene, achieving direct and synergistic cytotoxic effects, along with bystander cell killing. In this study, we constructed a semi-and pseudotyped-RRV (sp-RRV) system harboring two suicide genes—herpes simplex virus type 1 thymidine kinase (*TK*) and yeast cytosine deaminase (*CD*)*—*to verify the dissemination and antitumor efficacy of our sp-RRV system (spRRVe-sEF1α-*TK*/sRRVgp-sEF1α-*CD*) in seven patient-derived glioblastoma stem-like cells (GSCs). Flow cytometry and high-content analysis revealed a wide range of transduction efficiency and good correlation between the delivery of therapeutic genes and susceptibility to the prodrugs ganciclovir and 5-fluorocytosine in patient-derived GSCs in vitro. Intra-tumoral delivery of spRRVe-sEF1α-*TK*/sRRVgp-sEF1α-*CD*, combined with prodrug treatment, synergistically inhibited cell proliferation and angiogenesis while increasing apoptosis and the depletion of tumor-associated macrophages in orthotopic glioblastoma xenografts. Genomic profiling of patient-derived GSCs revealed that the key genes preventing sp-RRV infection and transmission were associated with cell adhesion, migration, development, differentiation, and proliferation. This is the first report demonstrating that a novel sp-RRV-mediated *TK*/*CD* double suicide gene transfer system has high oncolytic power against extremely heterogeneous and treatment-refractory glioblastomas.

## 1. Introduction

Glioblastoma is highly infiltrative and extremely heterogeneous at the genetic and molecular level, and harbors glioblastoma stem-like cells (GSCs) with unique features including self-renewal, cellular quiescence, and undifferentiated phenotype distinct from their differentiated, proliferative progeny that comprise the bulk tumor mass [1,2]. All these characteristics contribute to therapeutic failure, disease recurrence, tumor evolution, and poor prognosis [1,2,3,4], indicating the importance of targeting GSCs. Due to the low clinical forecast of existing standard glioblastoma cell lines such as U87, U251, and T98G, patient-derived GSCs and orthotopic xenografts that properly reflect the molecular, genetic, and organizational heterogeneity of parental tumors can predict the effectiveness and responsiveness of cancer drugs [1,2,3,4,5,6,7]. Integrative employment of comprehensive genomic profiling and short-term cultured patient-derived GSCs/orthotopic xenografts is critical for prioritizing therapeutic strategies for clinical studies and for predictive biomarker discovery toward more personalized approaches [3,4,5,6,7].

As glioblastomas are anatomically restricted and commonly recur locally, they are suitable for gene therapy, a recent technique with encouraging preclinical results that have led to clinical trials [8,9]. Inherently non-cytolytic retroviral replicating vectors (RRVs) from murine leukemia virus (MuLV) and gibbon ape leukemia virus (GaLV) [10,11] are advantageous for the transfer of suicide genes (owing to integration and replication only in rapidly dividing tumor cells and genetic stability) [12,13], a relatively straightforward method of construction that allows easy modification, and the induction of immunogenic cell death and bystander cytotoxic effects depends on a non-toxic pro-drug administration [9,13,14]. The most well-studied and extensively used suicide gene/prodrug systems against glioblastoma are the herpes simplex virus (HSV) type 1 thymidine kinase (*TK*)/ganciclovir (GCV) and yeast cytosine deaminase/5-fluorocytosine (*CD*/5-FC) systems [8,9,14,15,16]. Phosphorylation of GCV by TK induces accumulation of its cytotoxic metabolite, GCV triphosphate, leading to subsequent incorporation into DNA and apoptosis [17]. On the other hand, CD deaminates the prodrug 5-FC to form 5-FU that inhibits thymidylate synthase, resulting in depletion of deoxythymidine triphosphate (dTTP) pools, DNA double-strand breaks, and cell death [18]. For example, early clinical trials of Toca 511, an investigational gamma-RRV encoding *CD* in combination with subsequent oral extended-release 5-FC (Toca FC), showed a promising novel treatment for recurrent malignant high grade glioma [19,20,21].

Unfortunately, single gene transfer systems based on *CD* or *TK* are likely to initiate resistance to phosphorylated GCV and 5-FU in tumors [22,23,24,25], while the latter additionally suffers from poor prodrug activation [26] and limited cell toxicity [27]. Based on the distinct mechanisms of TK/GCV and CD/5-FC, dual gene transfer of *TK* and *CD* could achieve synergistic cell killing [15,28,29,30,31,32,33,34,35,36], which can be explained by the 5-FC mediated reduction of dTTP, which decreases deoxyguanosine triphosphate through allosteric regulation of ribonucleotide reductase, resulting in the increased incorporation of GCV triphosphate into DNA to increase cytotoxicity [15,34,37]. Therefore, a gene therapy vector system that can simultaneously express *TK* and *CD* in a cancer-specific manner will be advantageous for treating glioblastomas with inter-tumoral heterogeneity at genetic, proteomic, and epigenetic levels, which often leads to TK or CD resistance via the activation of alternative pathways [4,38].

Simultaneous insertion of *TK* and *CD* renders the RRV genome untenably large (≥10 kb) and the insertion of larger size therapy genes greatly increases the inefficiency of virus packaging and the re-combination of viral genomes [39]. The recently developed semi- and pseudotyped-RRV (sp-RRV) system, based on two trans-complementing replication-defective Moloney-murine leukemia viral (MuLV) vectors, can circumvent these obstacles [9,13,39]. Each of these vectors transduces a transgene and either *gag-pol* or *env*, which together contain all the necessary genetic material for replication and sp-RRV production upon transfection into a tumor cell [13,39]. The separation of the envelope-encoding transcriptional unit into independent transcriptional units offers considerable flexibility for envelope exchange, pseudotyping, use of genetically or chemically engineered envelope proteins, and change, restriction, or broadening of vector tropism [40]. This sp-RRV vector duo allows co-propagation of two different transgenes, which offers both a back-up therapeutic opportunity, should the effect of the first gene product wane owing to the development of drug resistance, and a means for vector replication shut off, if the transgene is a suicide gene [13].

**Figure 1:**
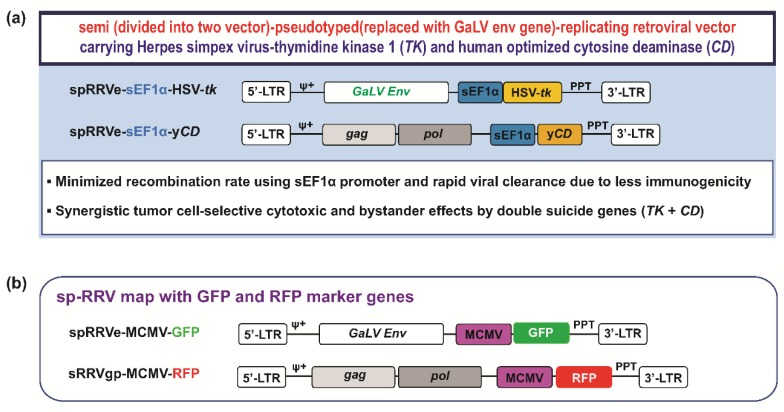
Schematic representation of a novel double-enhanced suicide gene delivery system using a semi-and pseudotyped-retroviral replicating vector (sp-RRV) system based on a gibbon ape leukemia virus (GaLV) carrying herpes simplex virus thymidine kinase (HSV-*tk*) and human codon-optimized yeast cytosine deaminase (y*CD*) for the treatment of glioblastoma. (**a**) A diagram illustrating spRRVe-short Elongation factor1-alpha (sEF1α)-*TK* and sRRVgp-sEF1α-*CD* systems. The sRRVgp vector contains the murine leukemia virus (MuLV) *gag*-*pol* coding sequence and the spRRVe vector was prepared by removing most of the MuLV *gag*-*pol* region and inserting the GaLV-*env* coding sequence. (**b**) A schematic vector diagram illustrating the structures of sp-RRV genome labeled with green fluorescence protein (GFP) (spRRVe-murine cytomegalovirus (MCMV)-GFP) and red fluorescence protein (RFP) (sRRVgp-MCMV-RFP) for the evaluation of virus infectivity. LTR: long terminal repeat.

Here, we investigated the transduction efficiency, anti-tumor efficacy, and bystander effects of this double suicide gene transfer based on a novel sp-RRV system with improved packaging efficiency of the oversized RNA genome and reduced possibility of losing therapeutic genes via recombination (Figure 1a, Patent: US 10039841) in a panel of patient-derived GSCs instead of standard glioblastoma cell lines. The first contained the *env* genes of GALV, a promoter elongation factor lα (EFlα) to confirm lack of recombination, and *TK* (spRRVe-sEF1α-*TK*). The second contained the MuLV *gag-pol*, a promoter, and human codon optimized yeast *CD* (sRRVgp-sEF1α-*CD*). This study first demonstrates inter-tumoral heterogeneity in gene delivery efficiency and the synergistic therapeutic effects of our sp-RRV mediated *TK*/*CD* dual gene transfer by applying patient-derived GSCs and orthotopic xenografts.

## 2. Results

### 2.1. Analysis of sp-RRV Dissemination In Vitro in Patient-Derived GSCs

We hypothesized that green fluorescent protein (GFP)- or red fluorescent protein (RFP)-only positive cells are equally important as the GFP/RFP dual-positive cells in assessing sp-RRV dissemination, as GSCs transduced with only one therapeutic gene (*TK* or *CD*) show considerable antitumor abilities owing to the enhanced bystander effects caused by phosphorylated GCV or 5-FU [8,9,14,23,41,42,43]; in such cases, these cytotoxic molecules can be delivered to the same tumor cells or nearby non-transduced tumor and tumor-promoting stromal cells. In addition, cells co-transduced with the sp-RRV system may be detected as GFP- or RFP-only positive cells in the case of predominant protein expression by only one vector.

**Figure 2:**
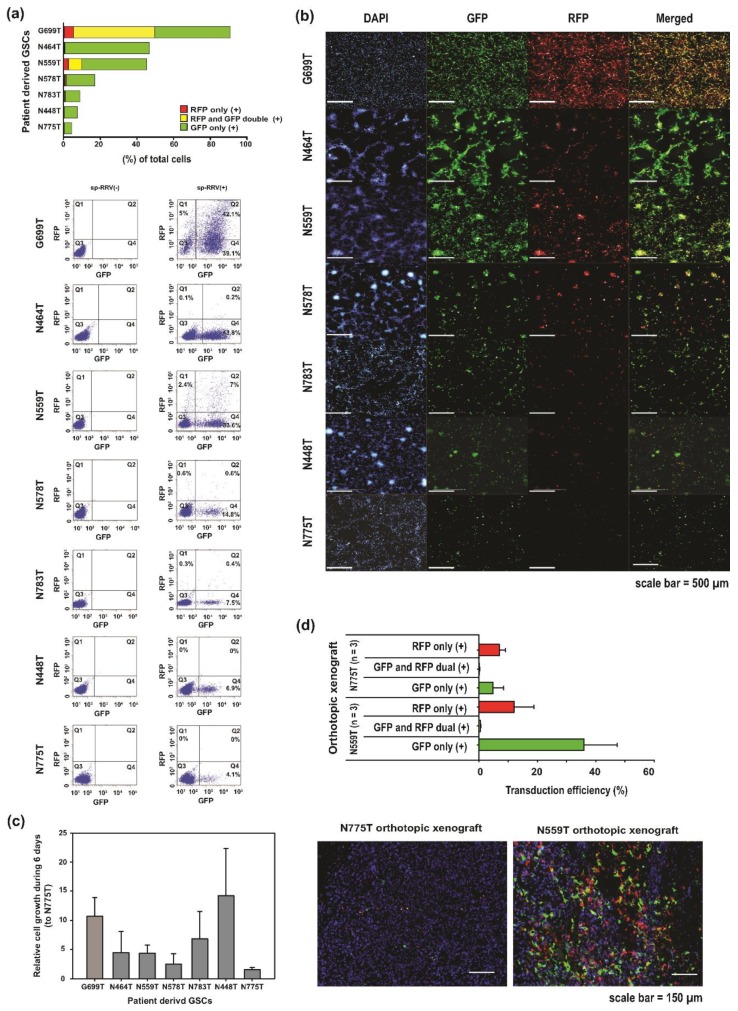
Patient-derived glioblastoma stem-like cells (GSCs) show diverse levels of susceptibility to sp-RRV system carrying spRRVe-GFP and sRRVgp-RFP vectors. (**a**) In vitro infectivity of sp-RRV carrying spRRVe-GFP and sRRVgp-RFP vectors in seven patient-derived GSCs. The graph indicates the percentage of GFP (single positive), RFP (single positive), and GFP and RFP (double-positive) cells as determined by fluorescence-activated cell sorting analysis on day 6 (Upper, bar plot; Lower, scatter plot). Each cell type was initially infected with spRRVe-GFP and sRRVgp-RFP vectors at a multiplicity of infection (MOI) of 1. (**b**) Representative immunofluorescence images of seven patient-derived GSCs infected with spRRVe-GFP and sRRVgp-RFP viral vectors. All GSCs were infected with spRRVe-GFP and sRRVgp-RFP for 1 week, and then analyzed by Operetta CLS High-Content Analysis System. The nuclei were counterstained with DAPI; scale bar = 500 µm. (**c**) In vitro cell proliferation of seven patient-derived GSCs during 6 days of incubation. Spheres were dissociated and seeded into 384-well plates at 500 cells per well in sphere media. During the 6 days culture period, cell viability was assayed by a one-step ATPlite adenosine triphosphate monitoring system based on firefly luciferase and estimated by EnVision Multilabel Reader (PerkinElmer, MA, USA) on day 0 (day after plating the cells) and 6. The graph shows the mean and standard error of the mean (SEM) of the viability ratio at day 6 and viability at day 0. The data represent mean and SEM of three independent experiments. (**d**) Validation of transduction efficiency of spRRVe-GFP and sRRVgp-RFP viral vectors in two patient-derived GSC (N775T and N559T)-based orthotopic xenografts. Whole brain tissues were embedded with optimal cutting temperature compound (#4583, Scigen, CA, USA), and images of two orthotopic xenografts (N775T and N559T) were analyzed by Vectra 3 automated quantitative pathology imaging system. The graph shows the fraction of GFP-, RFP-, and GFP/RFP double-positive cells evaluated by inForm Image Analysis Software. The results in the bar graph are shown as mean and SEM.

Based on this rationale, we defined transduction efficiency as the sum of the percentage of GFP, RFP, and dual-positive cells. Fluorescent activated cell sorting (FACS) (Figure 2a) and high-content analysis (Figure 2b) showed that among the seven patient-derived GSCs, G699T (86.1%), N464T (44.1%), and N559T (43%) showed better infectivity, while N448T and N775T showed the lowest infection rate (at 6.9 and 4.1%, respectively) on day 6. Interestingly, we did not observe any meaningful correlation between cell growth rate and sp-RRV permissivity (Figure 2c), which was consistent with the results of previous studies [7,22]. In a proof-of-concept study to correlate in vitro results with intra-tumoral dissemination, the in vivo transduction efficiency of sp-RRV harboring spRRVe-GFP and sRRVgp-RFP was superior in N559T orthotopic xenografts (GFP only, 56.7%; RFP only, 15.4%; GFP and RFP dual, 0.7%) than in N775T orthotopic xenografts (GFP only, 3.7%; RFP only, 9.9%; GFP and RFP dual, 0.2%) (Figure 2d).

### 2.2. Evaluation of 5-FC and GCV Sensitivity in Infected and Bystander Cells after TK and CD Delivery In Vitro

**Figure 3:**
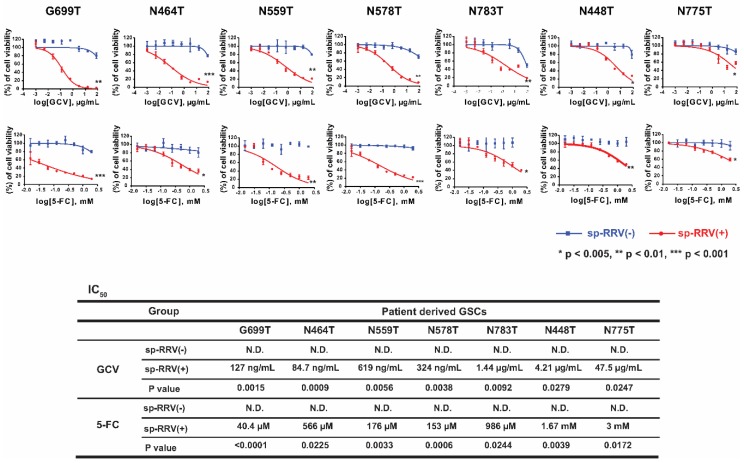
Different responses of seven patient-derived GSCs to GCV and 5-FC depend on the infectivity of spRRVe-sEF1α-*TK* and sRRVgp-sEF1α-*CD* viral vector systems. High-content screening for assessment of therapeutic efficacy of suicide genes (*TK* and *CD*) and two prodrugs (GCV, 0~78.125 µg/mL; 5-FC, 0~2 mM) in seven patient-derived GSCs treated with spRRVe-sEF1α-*TK* and sRRVgp-sEF1α-*CD* was performed. Nonlinear regression analyses of dose-response curves (upper panel) and half maximal inhibitory concentration (IC_50_) (lower panel) of seven patient-derived GSCs. Error bars represent standard deviation (SD). * *p* < 0.05, ** *p* < 0.01, *** *p* < 0.001.

High-content imaging is an inexpensive, rapid, and high-throughput tool for determining single cell phenotypes that allows researchers to assess the therapeutic efficacy and mechanisms of action of novel agents [44]. Next, we used high-content screening to verify the effects of the sp-RRV harboring the therapeutic genes. Cell viability markedly decreased after 5-FC and GCV treatment, indicating good viral dissemination in G699T, N464T, N559T, N578T, and N783T GSCs (Figure 3). Half maximal inhibitory concentration (IC_50_) values were not reached for most of the parental GSCs tested, even at 5-FC and GCV concentrations as high as 78 μg/mL and 2 mM, respectively. We observed that the five GSCs with good dissemination abilities (G699T, N464T, N559T, N578T, and N783T) were also highly sensitive to GCV treatment (IC_50_, 84.7 ng/mL~1.44 µg/mL) and 5-FC (IC_50_, 40 µM~986 µM), whereas cells in the group showing poor dissemination (N448T and N775T) were insensitive to GCV (IC_50_, 4.21 µg/mL~47.5 µg/mL) and 5-FC (IC_50_, 1.67 mM~3 mM) treatment, demonstrating good correlation between efficient delivery of therapeutic genes (*TK* and *CD*) via the sp-RRV system and ganciclovir/5-FC treatment [14,23].

### 2.3. Synergistic Anti-Tumor Effects of sp-RRVe-eEF1α-TK and sRRVgp-eEF1α-CD after Treatment with GCV and 5-FC in Glioblastoma Patient-Derived Orthotopic Xenografts

**Figure 4:**
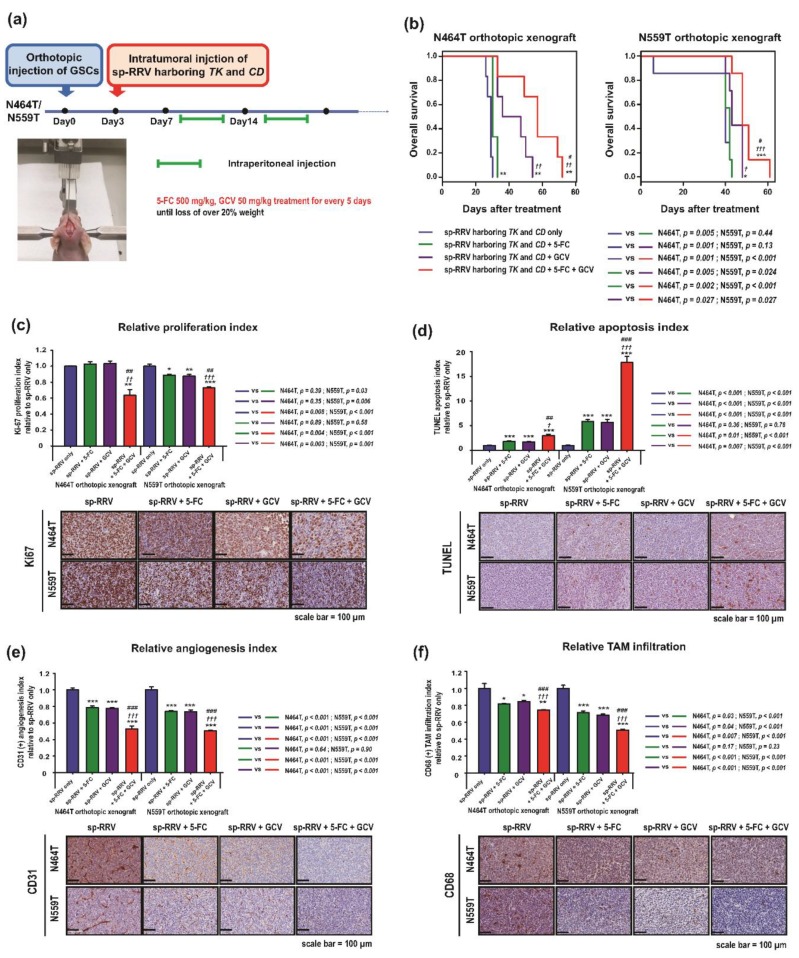
Anti-tumor effects, including inhibition of cell proliferation, induction of cell apoptosis, anti-angiogenesis, and depletion of tumor-associated macrophages (TAMs) of 5-FC combined with GCV in the spRRVe-sEF1α-*TK* and sRRVgp-sEF1α-*CD* sp-RRV vector systems are greater than those achieved using 5-FC or GCV alone. (**a**) Schematic diagram of the in-vivo experimental protocol. Blue and red arrows indicate orthotopic injection of two patient-derived GSCs (N464T and N559T) into BALB/c nude mice and intratumoral injection of spRRVe-sEF1α-*TK* and sRRVgp-sEF1α-*CD*, respectively. The long purple label represents intraperitoneal injection of PBS (*n* = 6), 5-FC (500 mg/kg, *n* = 6), GCV (50 mg/kg, *n* = 6), and a combination of 5-FC and GCV (*n* = 6), administered for 5 consecutive days/week until the mice were sacrificed. The mice were euthanized when there was more than 20% body weight loss. (**b**) Kaplan-Meier survival curves. (**c**,**d**) Analysis of proliferation index (Ki-67) (**c**) and apoptosis index (Terminal deoxynucleotidyl transferase dUTP nick end labeling (TUNEL) assay) (**d**) by immunohistochemistry (IHC). (**e**,**f**) IHC analysis of CD31 (**e**) and CD68 (**f**) expression in two orthotopic xenografts of patient-derived GSCs (N464T and N559T). Representative immunohistochemical staining images of Ki-67, TUNEL, CD31, and CD68 are shown. All the data are reported as the mean and SEM (**c**–**f**). (**b**–**f**) *, †, # *p* < 0.05, **, ††, ## *p* < 0.01, ***, †††, ### *p* < 0.001. * *p*-value vs. sp-RRV harboring TK and CD only group, † *p*-value vs. sp-RRV harboring TK and CD + 5-FC group, # *p*-value vs. sp-RRV harboring TK and CD + GCV group.

The infiltrative nature of glioblastoma restricts intratumoral distribution of the viral vector and impedes achievement of optimal clinical efficacy [38]. Till date, direct injection into the walls of the tumor resection cavity after surgical resection is the most commonly used strategy [41]. We next validated the efficacy of our novel dual suicide gene-delivery system in orthotopic xenografts established using N559T and N464T GSCs, which showed high infectivity by sp-RRV. After the orthotopic injection of N559T and N464T in nude mice, we re-delivered the spRRVe-sEF1α-*TK*/sRRVgp-sEF1α-*CD* into the same location to determine the efficacy of our approach as a novel adjuvant therapy against residual infiltrated glioblastoma tumor cells (Figure 4a). Furthermore, we used BALB/c nude mice with intact innate immune system, including monocytes/macrophages and natural killer cells, to determine whether administration of spRRVe-sEF1α-*TK*/sRRVgp-sEF1α-*CD* depleted the pro-tumoral tumor-associated macrophages (TAMs) via bystander effects [42].

Mice treated with 5-FC/GCV showed higher median survival (N464T, 57 days; N559T, 50 days) than control mice (N464T, 29 days, *p* = 0.001; N559T, 35 days, *p* < 0.001) (Figure 4b); this combination also elicited significantly longer survival than GCV or 5-FC alone (GCV: N464T, 36 days, *p* = 0.027; N559T, 42 days, *p* = 0.027; 5-FC: N464T, 30 days *p* = 0.002; N559T, 43 days, *p* < 0.001). Compared to either monotherapy or treatment with phosphate buffered saline (PBS), the combination of GCV or 5-FC considerably inhibited cellular proliferation (Figure 4c), increased apoptosis (Figure 4d), suppressed angiogenesis (Figure 4e), and enhanced TAM depletion (Figure 4f). In addition to the direct killing of tumor cells via production of intracellular phosphorylated GCV and 5-FU, the bystander anti-angiogenic and TAM depletion effects of nearby GSCs [8,9,14,23,41,43] might also contribute to these observations.

### 2.4. Determination of Host-Specific Inhibitory Factors to the sp-RRV System through Comprehensive Genomic Profiling of Patient-Derived GSCs

**Figure 5:**
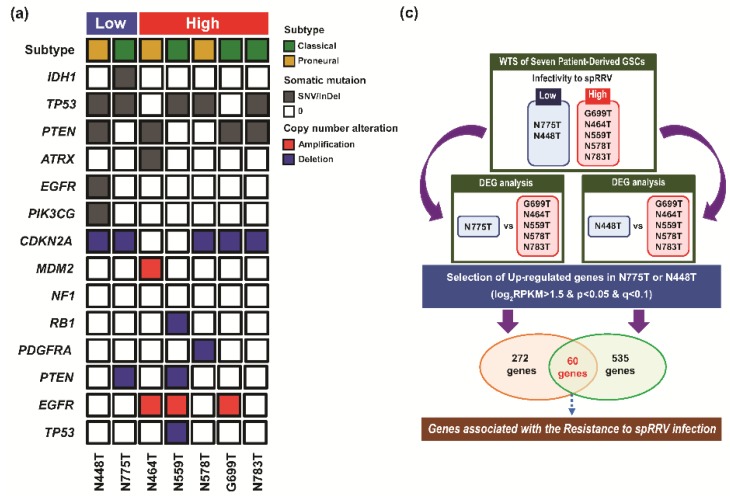

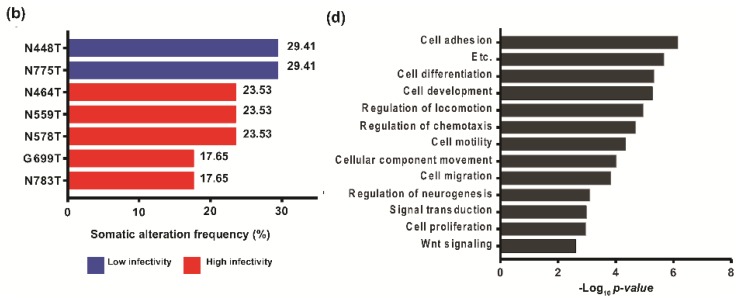
Analysis of host factors that inhibit infection with the sp-RRV system vector carrying *TK* and *CD* genes in patient-derived GSCs. (**a**) Summary of DNA sequencing results from tumors of seven patients with glioblastoma. Somatic mutational landscape of seven patient-derived GSCs, including single-nucleotide variants (SNVs), small insertions/deletions, and copy number alterations revealed by targeted-panel sequencing via GliomaSCAN™. Mutations are colored by the type of alteration, as indicated in the legend. (**b**) Somatic mutation frequency was calculated as the mutation frequency of 17 most frequently occurring genes. The bars of each row represent the frequency of SNVs and copy-number variation events found in each patient. (**c**) Schematic representation of host factors that inhibit infection with the sp-RRV system in seven patient-derived GSCs, as determined by differentially expressed gene (DEG) analysis of whole transcriptome sequencing (WTS). (**d**) Gene ontology (GO) was analyzed using the Database for Annotation, Visualization, and Integrated Discovery (DAVID); Bioinformatic Resources 6.8 was used to analyze the top 60 leading-edge genes identified by DEG analysis.

The development of new vectors that can overcome the limited infiltration and transgene expression occurring post-administration (due to host immunity to viral proteins and factors inhibiting virus-host interactions) are required to establish an optimally efficient therapeutic gene strategy [45]. For example, known cellular defense mechanisms are mainly responsible for inducing hypermutation via the apolipoprotein B mRNA editing enzyme catalytic subunit 3G, uncoating via tripartite motif-containing protein 5, and reduction of retrovirus replication and expression via various endogenous pathways [45]. To identify novel inhibitory factors for achieving optimal anti-cancer effects, we performed integrative genomic characterization (targeted-panel sequencing via GliomaSCAN™, whole transcriptome sequencing (WTS)) of the seven GSCs (categorized into high and low susceptibility to sp-RRV). Although infectivity did not show meaningful correlation with specific somatic alterations (Figure 5a), somatic alteration frequency was relatively higher in the two low-infectivity GSCs (N448T, N775T) than in high-infectivity GSCs (G699T, N464T, N559T, N578T, and N783T) (Figure 5b), consistent with previous observations showing that low DNA mutations forecast better response to Toca 511- and Toca FC-based therapy [20]. Next, to circumvent the limitation in the number of GSCs analyzed, we used differentially regulated gene (DEG) expression analysis with 60 genes that were relatively upregulated in each of the low-infectivity GSCs compared to in high-infectivity GSCs (Figure 5c and Table S1). Several Gene Ontology (GO) pathways, including “cell adhesion,” “cell development and differentiation,” “cell migration,” “cell proliferation,” and “Wnt signaling” were enriched in these genes associated with low susceptibility to infectivity with the sp-RRV system (Figure 5d).

## 3. Discussion

This is the first study to evaluate an sp-RRV-mediated *TK* and *CD* dual suicide gene delivery system in preclinical patient-derived GSCs, which is essential for investigating correlations between treatments and genotypes, while maximizing translation of promising agents into clinical settings [4,7]. Our sp-RRV system eliminated the chances of losing a therapeutic gene via recombination during viral infection. In addition, the GaLV envelope pseudotyping used in our system causes cell fusion, thereby increasing cytotoxicity and bystander effects, while preventing re-infection of vector particles [13] (Figure 1a, Patent: US 10039841). These characteristics reduce the likelihood of recombination and improve transduction via an increase in GaLV receptor expression on target cells [46,47]. We confirmed the versatility of our sp-RRV-mediated double suicide gene transfer system by investigating the preclinical antitumor efficacy and bystander effects of the combined vectors, spRRVe-sEF1α-*TK* and sRRVgp-sEF1α-*CD*, in seven patient-derived GSCs, each of which possessed unique properties (e.g., growth rates and genomic profiles). Our method synergistically halted cell division and induced cancer cell death, anti-angiogenesis, and TAM depletion in orthotopic patient-derived glioblastoma xenografts, which were consistent with the previously reported antitumor activities of the TK/GCV and CD/5-FC systems [8,9,14,23,38,48]. Similarly, recent studies have demonstrated that the fusion of TK and CD with GCV and 5-FC synergistically improves therapeutic efficacy via the enhancement of cytotoxicity and bystander effects [30,34,49].

Bystander effects depend on the prodrugs used and the mechanism via which the bystander cells acquire the activated cytotoxin [23,41]. TK can be transferred to neighboring cells via connexin gap-junctions or apoptotic vesicles released from infected dying cells, a process facilitated by cell-to-cell contact [23,41]. In contrast, 5-FU can diffuse to neighboring cells and cause bystander effects without any physical cell-cell contact [23]. In our combination therapy, noninfected neighboring cells may be killed via transfer of cytotoxic phosphorylated GCV and 5-FU from the nearby TK- or CD-expressing cells [43]. More importantly, GSCs can recruit tumor-associated non-tumor cells, such as reactive astrocytes, immune cells, and endothelial cells [50]. For example, TAMs, the dominant infiltrating population of immune cells, directly drive tumor growth by promoting immune suppression, cancer stemness, invasiveness, epithelial-mesenchymal transition, angiogenesis, and extracellular remodeling [50]. Local generation of 5-FU leads to ablation of myeloid-derived suppressor cells selectively within the tumor [43,51]. Based on these observations, we suggest that intercellular signaling and connections between GSCs, TAMs, and endothelial cells might amplify the overall therapeutic efficacy via the synergistic bystander effects of phosphorylated GCV and 5-FU.

GSCs with cellular quiescence and greater repair capacities than proliferative cells play a key role in the acquired or constitutive resistance to radio-chemotherapy [1,2]. As GaLV can only infect cells that are actively dividing [12], slow cycling patient-derived GSCs could be less susceptible to the sp-RRV system than their differentiated, proliferative progeny [1,2]. Notably, we found that sp-RRV dissemination occurred efficiently only in some GSCs. Heterogeneity in glioblastomas exists both between and within patients; variation in cancer-cell metabolism, signal transduction, and antiviral states alter the success of sp-RRV replication [52]. In order to achieve the optimal anti-cancer treatment effects despite the various benefits of retroviral vectors, a strategy must be developed to identify the inhibition mechanisms for replication of cancer immunity and other host-specific retroviruses, and to avoid such inhibition [45]. Importantly, we identified tripartite motif containing 62 (TRIM62) as a potential inhibitory factor, a TRIM family member that positively regulates apical-basal polarity and negatively regulates transforming growth factor-β-driven epithelial-mesenchymal transition [53]. Furthermore, genes involved in cell–cell adhesion, proliferation, and differentiation [54], such as partitioning defective 3 homolog [55], classical cadherin 13 [56], and members of the Wnt/β-catenin signaling pathway [57], were associated with low infectivity. Finally, cholesterol 25-hydroxylase, an enzyme that catalyzes oxidation of cholesterol to 25-hydroxycholesterol, can block viral infection via multiple mechanisms, including inhibiting viral envelope fusion with host membranes, viral replication, and formation of viral replication complexes on intracellular membranes [58]. Further studies validating and targeting these retroviral defense factors are required for developing patient-specific treatment strategies based on susceptibility of patient-derived cells/patient-derived xenografts and for circumventing major difficulties, including low efficiency of initial infection, rapid clearance of viral particles by innate immune cells, and physical barriers that limit particle dispersion.

The induction of antitumor immune responses after the initial killing of tumor cells is a potentially more powerful form of bystander effect [59]. These two responses may be indirectly related, because inflammation and the levels of released tumor antigens increase with GCV and 5-FU-mediated killing of tumor cells, leading to stronger antitumor immune response. For example, Toca 511/5-FC showed durable and complete response in immunogenic orthotopic glioma models in immune competent mice (Tu-2449-B6C3F1) by inducing local and systemic immunotherapeutic responses [59,60,61]. Therefore, the reasons for the lack of high levels of cure in our sp-RRV dual gene transfer may be the weak immunogenicity of patient-derived GSCs and the use of immune-deficient nude mice, consistent with previous studies showing that Toca 511 + 5-FC was not curative in Tu-2449 orthotopic models established in immunodeficient mice [62]. These results support dual mechanisms of action contributing to the efficacy of RRV-mediated prodrug-activator gene therapy: Long-term tumor control by prodrug conversion-mediated cytoreduction, and induction of long-term cellular antitumor immunity against native tumor antigens [51,61]. Depletion of immunosuppressive cells such as TAMs temporally preceded a second event which included the expansion of T cells which were polarized away from Th2 and Th17 in the CD4+ T cell compartment with concomitant expansion of interferon gamma–expressing CD8+ T cells [51,61]. It has also recently been reported that cytolytic CD4+ and CD8+ T cells can cooperatively attack retrovirus-infected cells [63]. Repeated cycles of intratumoral prodrug conversion by RRV not only achieves long-term disease control but, through local “bystander effects” on immunosuppressive tumor stroma, also permits development of durable cellular immune responses directed against endogenous tumor antigens, contributing to apparent complete eradication of residual disease [51,61]. Thus, there is an urgent need for further evaluation of our sp-RRV systems in patient-derived GSCs using humanized mouse models that have been developed to study the interactions between immune components and tumors of human origin through human immune cell reconstitution [64].

Furthermore, fine-tuning of our sp-RRV dual gene transfer strategy including rational treatment combinations and optimization of vector dose and prodrug scheduling should be performed to minimize failures in clinical trials. Previous studies in immunodeficient models have also indicated that long-term tumor control could be achieved but was dependent upon the continued administration of prodrug using a cyclic dosing schedule [61,65,66]. Additional combinations or regimens and cyclic prodrug dosing schedules may be required to break the immune tolerance and induce long-term immune memory by the intermittent release of tumor antigens. Recent studies demonstrated long-term survival advantage and complete remission of combinations of Toca 511/5-FC and the most frequently used treatments for patients with glioblastoma including temozolomide (TMZ) (in human glioma cell line U-87MG (TMZ-sensitive) orthotopic models) [67], radiation (in U87MG and its radioresistant variant U87EGFRvIII orthotopic models) [68] and lomustine (in immune-competent rat F98 and mouse Tu-2449 orthotopic models) [60], without preventing RRV spread nor interfere with Toca 511/5-FC-mediated cell killing. Finally, given recent interest in combined immuno-oncologic agents, the pro-immunogenic effects of RRV-mediated prodrug conversion for tumor cell killing and tumor antigen release and for bystander killing of immunosuppressive tumor stromal cells may prove widely useful.

## 4. Materials and Methods

### 4.1. Patient-Derived GSC Establishment

Human glioblastoma specimens were enzymatically dissociated into single cells within hours of surgical resection. All procedures were approved by the Samsung Medical Center (SMC) Institutional Review Board (IRB) (approval no. 2015-11-096) and conducted in accordance with the Declaration of Helsinki. Written informed consent for research-related use of tissues was obtained from patients when the tumor specimens were procured. The Samsung Medical Center BioBank provided the biospecimens. All experiments, including those involving sp-RRVs, were performed in authorized laboratories of the Samsung Medical Center (LML 16-824 for in vitro and LML 16-753 for in vivo, Chungnam National University: LML 16-610). All patient-derived GSCs were evaluated using short tandem repeats for cell authentication.

### 4.2. Sphere-Forming Culture of Patient-Derived GSCs

For in vitro expansion, GSCs were cultured in Neurobasal A media (NBA; 10888-022, Gibco, Waltham, MA, USA) supplemented with N2 and B27 (0.5× each; 17502-048 and 12587-010, Gibco), human recombinant basic fibroblast growth factor, and epidermal growth factor (20 ng/mL each; 233-FB-001MG/CF and 236-EG-01M, R&D Systems, Minneapolis, MN, USA), as well as 100× penicillin streptomycin-glutamine (10378-016, Gibco) [7]. Next, GSCs were cultured at 37 °C in a humidified incubator containing 5% CO_2_.

### 4.3. Drugs and Reagents

Respectively, ganciclovir and 5-fluorocytosine for both in vitro and in vivo assays were purchased from Roche (Cymevene^®^ vial, Basel, Switzerland) and Sigma-Aldrich (F7129, St. Louis, MO, USA).

### 4.4. sp-RRV Production

The *TK* and CD sp-RRV system was produced through transfecting 6 × 10^5^ 293T cells with 2.0 μg of spRRVe-sEF1α-*TK* and 2.0 μg of sRRVgp-sEF1α-*CD* plasmids; Lipofectamine (18324-012, Invitrogen, Carlsbad, CA, USA) and Plus reagents (11514-015) per manufacturer protocol. After 48 h, the supernatant was harvested, filtered through 0.45-μm syringe filters, and stored at −80 °C. Residual plasmid DNA was removed from viral supernatant using DNaseI from the Retrovirus Titer Set (#6166, TaKaRa Bio Inc., Shiga, Japan). Next, real-time PCR (RT-PCR) was performed in 25-μL reaction volume, with the following thermocycling schedule: 42 °C for 5 min; 95 °C for 10 s; 40 cycles of 95 °C for 5 s and 60 °C for 30 s; 95 °C for 15 s, 60 °C for 30 s, and 95 °C for 15 s. A reference curve of retrovirus copy number was prepared via plotting C_T_ (cycle threshold) values.

### 4.5. sp-RRV System Transduction of GSCs

Patient-derived GSC neurospheres were dissociated into single cells via a 1–3 min incubation at 37 °C after adding 1 mL Accutase in Dulbecco’s phosphate-buffered saline without Ca^2+^/Mg^2+^ Cell detachment solution (#AT-104, Innovative Cell Technologies, Inc., San Diego, CA, USA). At 12–18 h post-seeding, 5 × 10^5^ GSCs were transduced with spRRVe-MCMV-GFP and sRRVgp-MCMV-RFP at a multiplicity of infection (MOI) of 1 for one week.

### 4.6. Determination of the Transduction Efficiency of the Patient-Derived GSC sp-RRV System Using FACS Analysis and Operetta High-Content Imaging System In Vitro

To determine the efficiency of endogenous envelope expression within tumor cells, we used additional spRRVe-MCMV-GFP and sRRVgp-MCMV-RFP (Figure 1b). Transduction efficiency was calculated from the percentage of GFP, RFP, or GFP and RFP dual-positive (emits YFP signals) cells, indicative of sp-RRV infectivity and dissemination. To quantify efficiency, patient-derived GSCs were seeded at a density of 2 × 10^5^ cells per well in six-well plates. After 24 h, cells were transduced with spRRVe-MCMV-GFP and sRRVgp-MCMV-RFP at a MOI of 1. On day 6 post-infection, the cells were trypsinized, washed twice in PBS, and analyzed using a FACSCalibur flow cytometer (Becton Dickinson Biosciences, San Jose, CA, USA). Living cells expressing GFP, RFP, or YFP were quantified in pre-selected regions.

To verify GFP and RFP expression, GSCs were seeded at a density of 2 × 10^3^ cells per well in a 384-well plate (OptiPlate-384 Black Opaque 384-well Microplate, PerkinElmer, Waltham, MA, USA) and precoated with PBS-diluted laminin (1:100; L2020, Sigma-Aldrich). The cells were transduced with spRRVe-MCMV-GFP and sRRVgp-MCMV-RFP at a MOI of 1 the next day. On day 6 post-transduction, the cells were fixed with 4% paraformaldehyde in PBS at room temperature (RT) for 60 min, washed twice in PBS, and then stained with Hoechst 33342 in 0.15% Triton ×100 and 3% bovine serum albumin (BSA) in PBS for 60 min. The cells were washed again with PBS before imaging with an Operetta high-content imaging system (PerkinElmer) at 10× magnification and analyzed using the Harmony high-content analysis software (PerkinElmer).

### 4.7. In Vivo Transduction Efficacy of Orthotopic Xenografts Based on Patient-Derived GSCs and sp-RRV System

All in vivo experiments were conducted following guidelines from the Association for Assessment and Accreditation of Laboratory Animal Care of the Samsung Medical Center Animal Use and Care Committee (Assurance no. A16-004) and the National Institute of Health (Bethesda, Rockville, MD, USA) Guide for the Care and Use of Laboratory Animals (NIH publication 80-23). To establish orthotopic xenografts, 6-week-old female athymic BALB/c-nude mice were purchased from Orient Bio Inc. (Gyeonggi-do, Korea). Two patient-derived GSC cell lines (N775T and N559T) suspended at 2 × 10^5^ cells/5 μL in Hank’s Balanced Salt Solution (HBSS; 14170-112, Gibco) were directly injected into mouse brains using a rodent stereotactic frame (coordinates: anterior/posterior +0.5 mm, medial/lateral +1.7 mm, dorsal/ventral −3.2 mm). These coordinates were also used for injections of spRRVe-MCMV-GFP and sRRVgp-MCMV-RFP (3 × 10^7^ TU/5 μL) 1 week after GSC implantation. All mice were then sacrificed upon observing either 20% of total body weight loss, or neurological symptoms such as lethargy, ataxia, and seizures. Whole brains were extracted and flash frozen for cryosectioning. To determine sp-RRV spread and GFP/RFP spectra, sections were examined with VECTRA 3.0 Automated Quantitative Pathology Imaging System and InForm (PerkinElmer), respectively. Furthermore, DAPI staining was used to reveal individual nuclei within defined regions of tissue sections.

### 4.8. Cell Proliferation Assay

Patient-derived GSC-based screening and analysis followed published methods [7]. Seven patient-derived GSCs were dissociated to single cells and seeded in 384-well plates with duplicates (500 cells per well). On day 6, cell viability was analyzed using an ATP monitoring system based on firefly luciferase (ATPlite 1 step, PerkinElmer) and estimated in EnVision Multilabel Reader (PerkinElmer).

### 4.9. In Vitro GCV and 5-FC Sensitivity Assay of sp-RRV-Transduced Patient-Derived GSCs

Patient-derived GSCs were stained with CellEvent™ caspase-3/7 green detection reagent (1:2000, #C10423, Thermo Fisher, Waltham, MA, USA) and seeded at a density of 1 × 10^3^ cells per well in a 384-well plate (OptiPlate-384 Black, Black Opaque 384-well Microplate, PerkinElmer) precoated with PBS-diluted laminin (1:100; Sigma-Aldrich). On day 1, GSCs were transduced with spRRVe-sEF1α-*TK* and sRRVgp-sEF1α-*CD* at a MOI of 1. After 3 days, the cells were treated with GCV and 5-FC, at concentrations of 1 ng/mL to 78 μg/mL for the former and 15 μM to 2 mM for the latter after seven-point log-fold serial dilutions. At 72 h post-drug treatment, the cells were fixed with 4% paraformaldehyde and blocked for 1 h with PBS containing 1% BSA and 0.3% Triton X-100. At 5 days post-treatment, the cells were stained using Click-iT^®^ Plus EdU Alexa Fluor^®^ 594 imaging kits (# C10639, Thermo Fisher) and Hoechst33342 (1:5000), following the manufacturer’s protocol, before being washed with PBS. Next, cell images were obtained using Operetta at 10× magnification and analyzed using the Harmony high-content analysis software, following the manufacturer’s protocol (PerkinElmer). Nuclei of viable cells were counted per well and then grouped based on size and shape. Furthermore, distributions of total and viable cells, as well as percent cell viability, were determined. IC_50_ was determined using Prism 7 (GraphPad, San Diego, CA, USA). Caspase3/7 activity (indicator of apoptosis) was determined from the fluorescence intensity per cell using Cell Event caspase3/7 detection reagent (Thermo Fisher). Cells showing caspase 3/7-positive fluorescence (cut-off: 300–1000 AU depending on cell type) were considered dead and were excluded from total viable cell count. Data were collated from two or more individual experiments.

### 4.10. Validation of sp-RRV Therapeutic Efficacy in Orthotopic Xenografts

All in vivo experiments were conducted following the guidelines of the Association for Assessment and Accreditation of Laboratory Animal Care of the Samsung Medical Center Animal Use and Care Committee (Assurance no. A16-004) and the National Institute of Health (Bethesda) Guide for the Care and Use of Laboratory Animals (NIH publication 80-23). Two days after the tumors were induced in mice with N464T and N559T injections, the subjects were treated with intra-tumoral injections of the sp-RRV system (at same coordinates). Three days later, mice were randomly divided into four groups of six animals each. Group 1 was the control, which received PBS intraperitoneal injections. GCV (50 mg/mL/kg, once per day) only, 5-FC (500 mg/mL/kg, once per day) only, and 50 mg/mL/kg GCV plus 500 mg/mL/kg 5-FC were administered intraperitoneally using a cycle of 5 days on and 2 days off in Groups 2–4, respectively [18,69]. All treatments were administered until euthanasia. Mice with >20% weight loss for >2 days were euthanized; whole brains were extracted, formalin-fixed, and paraffin-embedded for further analysis.

### 4.11. Histological and IHC Analysis

Animals were sacrificed at different time points, and orthotopic tumor growth was analyzed after haematoxylin and eosin (H&E) staining of fixed brain tissues coronally sectioned at the thickness of 5 μm. Briefly, the sections were deparaffinized, rehydrated, stained with H&E, and scanned using Aperio AT Turbo Scanner (Leica Biosystems, Wetzlar, Germany). For IHC staining, sections were deparaffinized, rehydrated, and immersed in 3% hydrogen peroxide in methanol for 12 min to inactivate endogenous peroxidase. Then, sections were washed with PBS, blocked with 5% BSA (Gibco) in Dako REAL Peroxidase-Blocking Solution (S2023, Agilent, Santa Clara, CA, USA), and incubated using anti-Ki-67 (1:200, #9027S, Cell Signaling, Danvers, MA, USA), anti-CD31 (1:50, M0823, Agilent), and anti-CD68 (1:500, ab125212, Abcam, Cambridge, England) antibodies in antibody diluent with background (S3022, Agilent) and 5% BSA overnight at 4 °C. After incubation with the primary antibody, a mixed horseradish peroxidase-conjugated secondary antibody was applied onto the sections and allowed to incubate for 1 h at RT. Immuno-reactivity was visualized using diaminobenzidine tetrahydrochloride chromogenic substrate (Dako REAL™ EnVision™ Detection System, K5007, Agilent). Apoptosis extent per tumor was measured by TUNEL using a Calbiochem FragEL™ DNA Fragmentation Detection Kit and Colorimetric-TdT Enzyme (EMD Millipore, Burlington, MA, USA) following manufacturer’s protocol. Tissue sections were counterstained with hematoxylin, a coverslip was placed over the section, and sections were scanned using Aperio AT Turbo Scanner (Leica Biosystems).

### 4.12. Quantitative Analysis of IHC Staining

IHC images were captured with an automatic histologic imaging system (TissueFAXS, TissueGnostics GmbH, Vienna, Austria). The expression of Ki-67, TUNEL, CD31, and CD68 was quantified by HistoQuest Analysis Software using TissueFAXS system (TissueGnostics GmbH) after defining regions of interest. Several parameters, such as nuclei size and intensity of staining, were adjusted to achieve optimal cell detection. Cells were plotted to scattergrams according to human-specific marker signals. Cutoff thresholds were determined using signal intensity of the secondary antibody alone as negative control. Positive cell counts from images of immunohistolabeled sections were conducted by two independent observers blinded experimental conditions. The numbers of CD68-labeled tumor-associated macrophages (TAMs) were determined per 1 mm^2^ using HistoQuest Analysis Software using TissueFAXS system. The expression of CD68 receptor, which mediates the recruitment and activation of macrophages, is a marker for both monocytes and tissue macrophages [70]. Mean values for positive cells counted in five locations were evaluated. In areas with most intense CD31-positive neovascularization, micrographs were captured under ×200 magnification. Any endothelial cell or its cluster was considered as a single countable microvessel. The absolute number of quantified microvessels per area was considered as microvascular density.

### 4.13. Targeted-Panel Sequencing via GliomaSCAN™

Samples were profiled at the Samsung Medical Center using targeted-panel sequencing via GliomaSCAN™, a sequencing platform designed to target 312 genes specific for glioblastomas. An Agilent SureSelect kit was used to capture exonic DNA fragments. The Illumina HiSeq 2000 instrument (San Diego, CA, USA) was used to generate two 101 bp paired-end reads.

### 4.14. WTS

Total RNA from patient-derived GSCs was isolated with a RNeasy mini kit (#74106, Qiagen, Hilden, Germany) as recommended by the manufacturer’s protocol. For all samples, RNA-seq libraries were prepared from 500 ng total RNA using an Illumina TruSeq RNA Sample Prep kit. All sequenced reads were generated to include 30 nucleotides from the 5’ end of each read. After generating low-quality reads, we aligned them to the human reference genome (hg19) using GSNAP (version 2012-12-20) [71]. The resulting alignments were sorted and summarized into BED files using SAMtools and bedTools. BED files were used to calculate read values per kilobase of transcript per million reads (RPKM) for each gene using R package “DEGseq”.

### 4.15. DEG and GO Analysis of DEGs

DEGs were identified DEGseq, with the following cut-off threshold: |log_2_Fold change| > 1.5 and *p* ≤ 0.05, *q* ≤ 0.1. Seven samples were divided into two groups: susceptible versus insusceptible. The online Database for Annotation, Visualization, and Integrated Discovery (DAVID) was used for GO analysis of identified DEGs.

### 4.16. Statistical Analysis

Animal survival was plotted using the Kaplan–Meier method and long-rank tests were used for comparisons. Data are expressed as means and SEM or means and SD. Two-tailed t-tests or one-way analysis of variance (ANOVA) was performed whenever appropriate and *p*-values < 0.05 were considered significant. Data were analyzed using GraphPad Prism and SPSS version 16 (SPSS Inc., Chicago, IL, USA).

### 4.17. Data Access

All GliomaSCAN™ and whole transcriptome sequencing results have been deposited in the European Genome-Phenome Archive (EGA; http://www.ebi.ac.uk/ega/) hosted by the European Bioinformatics Institute under accession no. EGAS00001001041. As previously indicated [72], access to the deposited data is limited.

## 5. Conclusions

This is the first report investigating the therapeutic utility of a novel sp-RRV-mediated *TK*/*CD* double suicide gene transfer system against heterogeneous patient-derived GSC models by demonstrating effective dissemination and synergistic anti-tumor effects compared to monotherapies.

## Supplementary Materials

The following are available online at https://www.mdpi.com/2072-6694/11/8/1090/s1, Table S1: Genes associated with the resistance to sp-RRV harboring *TK* and *CD*.
