# Supplementary material for: Novel Semi-Replicative Retroviral Vector Mediated Double Suicide Gene Transfer Enhances Antitumor Effects in Patient-Derived Glioblastoma Models
Source: Cancers (Basel). 2019 Jul 31;11(8):1090. doi: 10.3390/cancers11081090 (PMC6721803; doi:10.3390/cancers11081090)
Supplement: Supplementary file 1 [file cancers-11-01090-s001.pdf]

Supplementary Materials

Novel Semi-Replicative Retroviral Vector Mediated Double Suicide Gene Transfer Enhances Antitumor Effects in Patient-Derived Glioblastoma Models

Mijeong Lee, Yeon-Soo Kim, Kyoungmin Lee, Moonkyung Kang, Hyemi Shin, Jeong-Woo Oh, Harim Koo, Donggeon Kim, Yejin Kim, Doo-Sik Kong, Do-Hyun Nam and Hye Won Lee

Materials and Methods

1. Drugs and Reagents

Respectively, ganciclovir and 5-fluorocytosine for both in vitro and in vivo assays were purchased from Roche (Cymevene® vial, Basel, Switzerland) and Sigma-Aldrich (F7129, MO, USA).

2. Sphere-Forming Culture of Patient-Derived GSCs

For in vitro expansion, GSCs were cultured in Neurobasal A media (NBA; 10888-022, Gibco, MA, USA) supplemented with N2 and B27 (0.5× each; 17502-048 and 12587-010, Gibco, MA, USA), human recombinant basic fibroblast growth factor (bFGF), and epidermal growth factor (20 ng/mL each; 233-FB-001MG/CF and 236-EG-01M, R&D Systems, MN, USA), as well as 100× penicillin streptomycin-glutamine (10378-016, Gibco, MA, USA) [1]. Next, GSCs were cultured at 37 °C in a humidified incubator containing 5% CO_2.

3. Cell Proliferation Assay

Patient-derived glioblmastoma stem-like cell (GSC)-based screening and analysis followed published methods.[1] Seven patient-derived GSCs were dissociated to single cells and seeded in 384-well plates with duplicates (500 cells per well). On day 6, cell viability was analyzed using an ATP monitoring system based on firefly luciferase (ATPlite 1 step, PerkinElmer, MA, USA) and estimated in EnVision Multilabel Reader (PerkinElmer, MA, USA).

4. sp-RRV Production

The *TK* and *CD* sp-RRV system was produced through transfecting 6 × 10^5 293T cells with 2.0 µg of spRRVe-sEF1α-*TK* and 2.0 µg of sRRVgp-sEF1α-*CD* plasmids; Lipofectamine (18324-012, Invitrogen, MA, USA) and Plus reagents (11514-015, Invitrogen) per manufacturer protocol. After 48 h, the supernatant was harvested, filtered through 0.45-µm syringe filters, and stored at −80 °C. Residual plasmid DNA was removed from viral supernatant using DNaseI from the Retrovirus Titer Set (#6166, TaKaRa Bio Inc., Shiga, Japan). Next, real-time PCR (RT-PCR) was performed in 25-µL reaction volume, with the following thermocycling schedule: 42 °C for 5 min; 95 °C for 10 s; 40 cycles of 95 °C for 5 s and 60 °C for 30 s; 95 °C for 15 s, 60 °C for 30 s, and 95°C for 15 s. A reference curve of retrovirus copy number was prepared via plotting C_T (cycle threshold) values.

5. sp-RRV System Transduction of GSCs

Patient-derived GSC neurospheres were dissociated into single cells via a 1–3 min incubation at 37 °C after adding 1 mL Accutase in Dulbecco's phosphate-buffered saline without Ca^{2+}/Mg^{2+} Cell detachment solution (#AT-104, Innovative Cell Technologies, Inc., CA, USA). At 12–18 h post-seeding, 5 × 10^5 GSCs were transduced with sp-RRVe-murine cytomegalovirus (MCMV) immediate-early promoter (spRRVe-MCMV-GFP), and red fluorescent protein (sRRVgp-MCMV-RFP) at a multiplicity of infection (MOI) = 1 for one week.

6. In Vivo Transduction Efficacy of Orthotopic Xenografts Based on Patient-Derived GSCs and sp-RRV System

All in vivo experiments were conducted following guidelines from the Association for Assessment and Accreditation of Laboratory Animal Care of the Samsung Medical Center Animal Use and Care Committee (Assurance no. A16-004) and the National Institute of Health (Bethesda, MD, USA) Guide for the Care and Use of Laboratory Animals (NIH publication 80-23). To establish orthotopic xenografts, 6-week-old female athymic BALB/c-nude mice were purchased from Orient Bio Inc. (Gyeonggi-do, South Korea). Two patient-derived GSC cell lines (N775T and N559T) suspended at 2×10^5 cells/5 μL in Hank's Balanced Salt Solution (HBSS; 14170-112, Gibco, MA, USA) were directly injected into mouse brains using a rodent stereotactic frame (coordinates: anterior/posterior +0.5 mm, medial/lateral +1.7 mm, dorsal/ventral −3.2 mm). These coordinates were also used for injections of spRRVe-MCMV-GFP and sRRVgp-MCMV-RFP (3×10^7 TU/5 μL) 1 week after GSC implantation. All mice were then sacrificed upon observing either 20% of total body weight loss, or neurological symptoms such as lethargy, ataxia, and seizures. Whole brains were extracted and flash frozen for cryosectioning. To determine sp-RRV spread and GFP/RFP spectra, sections were examined with VECTRA 3.0 Automated Quantitative Pathology Imaging System and InForm (PerkinElmer, MA, USA), respectively. Furthermore, DAPI staining was used to reveal individual nuclei within defined regions of tissue sections.

7. Histological and Immunohistochemical (IHC) Analysis

Animals were sacrificed at different time points, and orthotopic tumor growth was analyzed after H&E staining of fixed brain tissues coronally sectioned at the thickness of 5 μm. Briefly, the sections were deparaffinized, rehydrated, stained with HE, and scanned using Aperio AT Turbo Scanner (Leica Biosystems, Wetzlar, Germany). For IHC staining, sections were deparaffinized, rehydrated, and immersed in 3% hydrogen peroxide in methanol for 12 min to inactivate endogenous peroxidase. Then, sections were washed with PBS, blocked with 5% BSA (Gibco) in Dako REAL Peroxidase-Blocking Solution (S2023, Agilent, CA, USA), and incubated using anti-Ki-67 (1:200, #9027S, Cell Signaling, MA, USA), anti-CD31 (1:50, M0823, Agilent, CA, USA), and anti-CD68 (1:500, ab125212, Abcam, Cambridge, England) antibodies in antibody diluent with background (S3022, Agilent, CA, USA) and 5% BSA overnight at 4 °C. After incubation with the primary antibody, a mixed horseradish peroxidase-conjugated secondary antibody was applied onto the sections and allowed to incubate for 1 h at RT. Immuno-reactivity was visualized using diaminobenzidine tetrahydrochloride chromogenic substrate (Dako REAL™ EnVision™ Detection System, K5007, Agilent, CA, USA). Apoptosis extent per tumor was measured by TUNEL using a Calbiochem FragEL™ DNA Fragmentation Detection Kit and Colorimetric-TdT Enzyme (EMD Millipore) following manufacturer's protocol. Tissue sections were counterstained with hematoxylin, a coverslip was placed over the section, and sections were scanned using Aperio AT Turbo Scanner (Leica Biosystems).

8. Quantitative Analysis of IHC Staining

IHC images were captured with an automatic histologic imaging system (TissueFAXS, TissueGnostics GmbH, Vienna, Austria). The expression of Ki-67, TUNEL, CD31, and CD68 was quantified by HistoQuest Analysis Software using TissueFAXS system (TissueGnostics) after defining regions of interest. Several parameters, such as nuclei size and intensity of staining, were adjusted to achieve optimal cell detection. Cells were plotted to scattergrams according to human-specific marker signals. Cutoff thresholds were determined using signal intensity of the secondary antibody alone as negative control. Positive cell counts from images of immunohistolabeled sections were conducted by two independent observers blinded experimental conditions. The numbers of CD68-labeled tumor-associated macrophages (TAMs) were determined per 1 mm^2 using HistoQuest Analysis Software using TissueFAXS system. The expression of CD68 receptor, which mediates the recruitment and activation of macrophages, is a marker for both monocytes and tissue macrophages

[2]. Mean values for positive cells counted in five locations were evaluated. In areas with most intense CD31-positive neovascularization, micrographs were captured under ×200 magnification. Any endothelial cell or its cluster was considered as a single countable microvessel. The absolute number of quantified microvessels per area was considered as microvascular density.

9. Targeted-Panel Sequencing via GliomaSCAN™

Samples were profiled at the Samsung Medical Center using targeted-panel sequencing via GliomaSCAN™, a sequencing platform designed to target 312 genes specific for glioblastomas. An Agilent SureSelect kit (CA, USA) was used to capture exonic DNA fragments. The Illumina HiSeq 2000 instrument (CA, USA) was used to generate two 101 bp paired-end reads.

10. Whole Transcriptome Sequencing

Total RNA from patient-derived GSCs was isolated with a RNeasy mini kit (#74106, Qiagen, Hilden, Germany) as recommended by the manufacturer's protocol. For all samples, RNA-seq libraries were prepared from 500 ng total RNA using an Illumina TruSeq RNA Sample Prep kit. All sequenced reads were generated to include 30 nucleotides from the 5′ end of each read. After generating low-quality reads, we aligned them to the human reference genome (hg19) using GSNAP (version 2012-12-20) [3]. The resulting alignments were sorted and summarized into BED files using SAMtools and bedTools. BED files were used to calculate read values per kilobase of transcript per million reads (RPKM) for each gene using R package "DEGseq."

11. Determination of Differentially Expressed Gene (DEG) and Gene Ontology (GO) Analysis of DEGs

DEGs were identified DEGseq, with the following cut-off threshold: $|\log_2 \text{Fold change}| > 1.5$ and p value ≤ 0.05, q value ≤ 0.1. Seven samples were divided into two groups: susceptible versus insusceptible. The online Database for Annotation, Visualization, and Integrated Discovery (DAVID) was used for GO analysis of identified DEGs.

References

1. Lee, J.K.; Liu, Z.; Sa, J.K.; Shin, S.; Wang, J.; Bordyuh, M.; Cho, H.J.; Elliott, O.; Chu, T.; Choi, S.W.; et al. Pharmacogenomic landscape of patient-derived tumor cells informs precision oncology therapy. *Nat. Genet.* **2018**, *50*, 1399–1411.
2. Guadagno, E.; Presta, I.; Maisano, D.; Donato, A.; Pirrone, C.K.; Cardillo, G.; Corrado, S.D.; Mignogna, C.; Mancuso, T.; Donato, G.; et al. Role of Macrophages in Brain Tumor Growth and Progression. *Int. J. Mol. Sci.* **2018**, *19*.
3. Wu, T.D.; Nacu, S. Fast and SNP-tolerant detection of complex variants and splicing in short reads. *Bioinformatics* **2010**, *26*, 873–881.

Table S1. Genes associated with the resistance to sp-RRV harboring *TK* and *CD.*

Gene Name	Protein Name	Gene Location	Function
AMOTL1	Angiomotin-like protein 1	11q21	Inhibits the Wnt/beta-catenin signaling pathway by recruiting CTNNB1 to recycling endosomes
CACNG7	Voltage-dependent calcium channel gamma-7 subunit	19q13.42	Regulates the activity of L-types calcium channels that contain CACNA1C as pore-forming subunit<br>Regulates the trafficking and gating properties of AMPA-selective glutamate receptors (AMPARs)
CALCRL	Calcitonin gene-related peptide type I receptor	2q32.1	Receptor for calcitonin-gene-related peptide (CGRP) together with RAMP1 and receptor for adrenomedullin together with RAMP3
CBLN1	Cerebellin-1	16q12.1	Required for synapse integrity and synaptic plasticity<br>Induces accumulation of synaptic vesicles in the pre-synaptic part by binding
CDH13	Cadherin-13	16q23.3	Calcium-dependent cell adhesion proteins
CDH15	Cadherin-15		
CH25H	Cholesterol 25-hydroxylase	10q23.31	Catalyzes the formation of 25-hydroxycholesterol from cholesterol<br>Plays an important role in regulating lipid metabolism by synthesizing a corepressor that blocks sterol regulatory element binding protein processing
COL5A2	Collagen alpha-2(V) chain	2q32.2	A member of group I collagen<br>Binds to DNA, heparan sulfate, thrombospondin, heparin and insulin
CPNE4	Copine-4	3q22.1	Calcium-dependent phospholipid-binding protein that plays a role in calcium-mediated intracellular processes
CXCL12	Stromal cell-derived factor 1	10q11.21	Activates CXCR4 to induces a rapid and transient rise in the level of intracellular calcium ions and chemotaxis<br>Binds to atypical chemokine receptor ACKR3, which activates the beta-arrestin pathway
DRD2	D(2) dopamine receptor	11q23.2	Dopamine receptor whose activity is mediated by G proteins which inhibit adenylyl cyclase
FAM131B	Protein FAM131B	7q34	
FAT3	Protocadherin Fat 3	11q14.3	Play a role in the interactions between neurites derived from specific subsets of neurons during development
FBLIM1	Filamin-binding LIM protein 1	1p36.21	Implicated in cell shape modulation and motility by serving as an anchoring site for cell-ECM adhesion proteins and filamin-containing actin filaments
FCRLA	Fc receptor-like A	1q23.3	Implicated in B-cell differentiation and lymphomagenesis
FLRT2	Leucine-rich repeat transmembrane protein FLRT2	14q31.3	Function in cell-cell adhesion, cell migration and axon guidance
FOXF2	Forkhead box protein F2	6p25.3	Transcription activator for a number of lung-specific genes
FZD8	Frizzeled-8	10p11.21	Receptor for Wnt proteins
H1F0	Histone H1.0	22q13.1	Necessary for the condensation of nucleosome chains into higher-order structures

Gene Name	Protein Name	Gene Location	Function
			Found in cells that are in terminal stages of differentiation or that have low rates of cell division
IGSF21	Immunoglobulin superfamily member 21	1p36.13	Selectively regulates inhibitory presynaptic differentiation through interacting with presynaptic NRXN2
ITGA2	Integrin alpha-2	5q11.2	A receptor for laminin, collagen, collagen C-propeptides, fibronectin and E-cadherin
JAG1	Protein jagged-1	20p12.2	Ligand for multiple Notch receptors and involved in Notch signaling
KCNJ6	G protein-activated inward rectifier potassium channel 2	21q22.13	Allows potassium to flow into the cell
KDR	Vascular endothelial growth factor receptor 2	4q12	Promotes proliferation, survival, migration and differentiation of endothelial cells
KIRREL3	Kin of IRRE-like protein 3	11q24.2	Homophilic adhesion molecule that promotes trans cellular interactions and stabilize mossy fiber filipodia contact and subsequent synapse formation
KLHL13	Kelch-like protein 13	Xq24	Substrate-specific adapter of a BTB-CUL3-RBX1 E3 ubiquitin-protein ligase complex required for mitotic progression and cytokinesis
LPHN2	Adhesion G proteincoupled receptor L2	1p31.1	Calcium-independent receptor of low affinity for alpha-latrotoxin
MIDN	Midnolin	19q13.3	Facilitates ubiquitin-independent degradation of polycomb protein CBX4
MIR4661	MicroRNA 4661	5q31.1	
NEURL1B	E3 ubiquitin-protein ligase NEURL1B	5q35.1	E3 ubiquitin-protein ligase involved in regulation of the Notch pathway through influencing the stability and activity of several Notch ligands
NEUROD1	Neurogenic differentiation factor 1	2q31.3	Associates with the p300/CBP transcription coactivator complex to stimulate transcription of the secretin gene as well as the gene encoding the CDKN1A
NOS2	Nitric oxide synthase, inducible	17q11.2	Involved in inflammation, enhances the synthesis of proinflammatory mediators such as NO, IL6 and IL8
NRP1	Neuropillin-1	10p11.22	Mediates the chemorepulsant activity of semaphorins<br>Coexpression with KDR results in increase VEGF 165 binding to KDR as well as increased chemotaxis
NUAK1	NUAK family SNF1-like kinase 1	12q23.3	Serine/threonine-protein kinase involved in various processes such as cell adhesion, regulation of cell ploidy, DNA damage response, senescence, cell proliferation and tumor progression
ODZ2	Teneurin-2	5q34	Induces homophilic cell-cell adhesion as a cellular signal transducer
ODZ3	Teneurin-3	4q34.3-q35.1	Involved in neural development by regulating the establishment of proper connectivity within the nervous system
PARD3	Partitioning defective 3 homolog	10p11.22-p11.21	Adapter protein involved in asymmetrical cell division and cell polarization

Gene Name	Protein Name	Gene Location	Function
PCDHB5	Protocadherin beta-5	5q31.3	Potential calcium-dependent cell-adhesion protein
PCDHB10	Protocadherin beta-10		
PRSS12	Neurotrypsin	4q26	Plays a role in neuronal plasticity and the structural reorganizations associated with learning and memory operations
PTGFRN	Prostaglandin F2 receptor negative regulator	1p13.1	Inhibits the binding of prostaglandin F2-alpha to its specific FP receptor
RCAN3	Calcipressin-3	1p36.11	Inhibits calcineurin-dependent transcriptional responses by binding to the catalytic domain of calcineurin A
ROBO2	Roundabout homolog 2	3p12.3	Receptor for SLIT2, and probably SLIT1, which are thought to act as molecular guidance cue in cellular migration
SLC27A3	Long-chain fatty acid transport protein 3	1q21.3	Has acyl-CoA ligase activity for long-chain and very-long-chain fatty acids
SNAI2	Zinc finger protein SNAI2	8q11.21	Plays an essential role in TWIST1-induced EMT and its ability to promotes invasion and metastasis
SPOCK2	Testican-2	10q22.1	Participates in diverse steps of neurogenesis by binding calcium
SPON1	Spondin-1	11p15.2	Cell adhesion protein that promotes the attachment of spinal cord and sensory neuron cells and the outgrowth of neurites
TCF7L1	Transcription factor 7-like 1	2p11.2	Participates in the Wnt signaling pathway<br>Acts as a repressor in the absence of CTNNB1, and as an activator in its presence
TCF7L2	Transcription factor 7-like 2	10q25.2-q25.3	A high mobility group box-containing transcription factor that plays a key role in the Wnt signaling pathway
TLE4	Transducin-like enhancer protein 4	9q21.31	Inhibits the transcriptional activation mediated by PAX5, and by CTNNB1 and TCF family members in Wnt signaling
TMEM 178A	Transmembrane protein 178A	2p22.1	Acts as a negative regulator of osteoclast differentiation in basal and inflammatory conditions by regulating TNFSF11-induced Ca fluxes
TP73	Tumor protein p73	1p36.32	Participates in the apoptotic responses to DNA damage
TRIM62	E3 ubiquitin-protein ligase TRIM 62	1p35.1	E3 ubiquitin ligase whose activity is dependent on E2 ubiquitin-conjugating enzyme UBE2D2
TSHZ3	Teashirt homolog 3	19q12	Transcriptional regulator involved in developmental processes association with APBB1, SET and HDAC factors as a transcriptional repressor
TUBA4A	Tubulin alpha-4A chain	2q35	Major constituent of microbules
VMAC	Vimentin-type intermediate filament-associated coiled-coil protein	19p13.3	
VWC2	Brorin	7p12.2	BMP antagonist playing a role in neural development and promoting cell adhesion

Gene Name	Protein Name	Gene Location	Function
WSCD2	WSC domain-containing protein 2	12q23.3	
ZDHHC23	Palmitoyltransferase DHHC23	3q13.31	Palmitoyltransferase that mediates palmitoylation of KCNMA1 involved in NOS1 regulation and targeting to the synaptic membrane
ZNF518B	Zinc finger protein 518B	4p16.1	Involved in transcriptional regulation

© 2019 by the authors. Licensee MDPI, Basel, Switzerland. This article is an open access article distributed under the terms and conditions of the Creative Commons Attribution (CC BY) license (http://creativecommons.org/licenses/by/4.0/).
